# Docosahexaenoic Acid Attenuates Mitochondrial Alterations and Oxidative Stress Leading to Cell Death Induced by Very Long-Chain Fatty Acids in a Mouse Oligodendrocyte Model

**DOI:** 10.3390/ijms21020641

**Published:** 2020-01-18

**Authors:** Thomas Nury, Margaux Doria, Gérard Lizard, Anne Vejux

**Affiliations:** Team ‘Biochemistry of the Peroxisome, Inflammation and Lipid Metabolism’ EA 7270, Université de Bourgogne Franche-Comté/Inserm, 21000 Dijon, France; thomas.nury@u-bourgogne.fr (T.N.); margaux.doria@hotmail.fr (M.D.); gerard.lizard@u-bourgogne.fr (G.L.)

**Keywords:** very long-chain fatty acid, lipotoxicity, oligodendrocytes, docosahexaenoic acid

## Abstract

In the case of neurodegenerative pathologies, the therapeutic arsenal available is often directed towards the consequences of the disease. The purpose of this study is, therefore, to evaluate the ability of docosahexaenoic acid (DHA), a molecule present in certain foods and considered to have health benefits, to inhibit the cytotoxic effects of very long-chain fatty acids (C24:0, C26:0), which can contribute to the development of some neurodegenerative diseases. The effect of DHA (50 µM) on very long-chain fatty acid-induced toxicity was studied by several complementary methods: phase contrast microscopy to evaluate cell viability and morphology, the MTT test to monitor the impact on mitochondrial function, propidium iodide staining to study plasma membrane integrity, and DHE staining to measure oxidative stress. A Western blot assay was used to assess autophagy through modification of LC3 protein. The various experiments were carried out on the cellular model of 158N murine oligodendrocytes. In 158N cells, our data establish that DHA is able to inhibit all tested cytotoxic effects induced by very long-chain fatty acids.

## 1. Introduction

In some neurodegenerative diseases, very long-chain fatty acids (VLCFA, carbon atoms > 22) are present in abnormally large quantities in many tissues and can induce lipotoxicity. Fatty acids can be provided by food or result from dysfunction of organelles. A disorder of β-oxidation, which occurs in the peroxisome, results in an increase in VLCFA concentration. VLCFA status is expressed as the ratio between the levels of C26:0 (hexacosanoic acid or cerotic acid) and C22:0 (docosanoic acid or behenic acid). Most hereditary peroxisomal disorders result in an increase in VLCFA. Alterations in peroxisomal function are also suspected of contributing in the etiology of various neurodegenerative diseases: (a) multiple sclerosis: high concentrations of C26:0 are present in grey matter as well as in serum [1,2], (b) Alzheimer’s disease: there is an accumulation of C22:0, C24:0 (tetracosanoic acid or lignoceric acid) and C26:0 [3], (c) X-linked adrenoleukodystrophy (X-ALD): there is an accumulation of C24:0, C26:0 and C26:1, in tissue and plasma caused by mutations in the ABCD1 gene, (d) dementia: there are higher levels of C26:0 in the plasma and red blood cells [4]. In these different pathologies, the central and/or peripheral nervous system is affected and undergoes demyelination. Oligodendrocytes are the cells that synthesize myelin and are therefore impacted by the lipotoxicity of VLCFA. Our laboratory and others have shown that VLCFA induces cell death associated with changes in plasma membrane, mitochondrial activity, oxidative stress, and late apoptosis. Recently, we have shown that cells utilize autophagy to protect themselves from VLCFA-induced lipotoxicity [5]. Oxidative stress, being a common element in different neurodegenerative diseases, has been the target for therapies. For example, the Aurora Pujol’s team tested an antioxidant cocktail: N-acetyl-cysteine, α-lipoic acid, and α-tocopherol in an X-linked adrenoleukodystrophy model [6]. This cocktail is capable of reversing the oxidative stress and locomotor impairment induced by VLCFA [6,7]. These authors also highlighted other less conventional antioxidants such as resveratrol, or mitochondrial biogenesis boosters, such as pioglitazone [8]. With the work of Pujol et al. and of our team, it has been shown that autophagy can also be a target, with the use of temsirolimus or rapamycin [5,8]. As a new therapeutic possibility, we have chosen to test a molecule that can be provided by food and is present in high amounts in blue fishes, which are major components of the Mediterranean diet; this molecule is docosahexaenoic acid (DHA: C22:6 n-3). We chose this molecule because DHA is known to play multi-functional roles in brain health and disease, and because numerous studies have proposed its use as a nutraceutical due to its neuroprotective effects. Modifications in fatty acid metabolism leading to endogenous synthesis of DHA may also be involved in the development of certain pathologies such as those related to aging or neurodegenerative diseases. Indeed, DHA brain levels are decreased in the peroxisomal disease Zellweger syndrome [9] and in an Alzheimer’s disease model (human neuronal SK-N-BE cells). In addition, DHA can also be involved in the phenomena of oxidative stress and cell death, as shown in a model of Alzheimer’s disease. In the SK-N-BE cell line, DHA (at a concentration of 50 μM) attenuates mitochondrial dysfunction and oxidative stress [10]. In this study, we evaluated the effect of DHA on different parameters related to cell death in 158N murine oligodendrocytes: cell viability, mitochondrial activity, membrane permeability, oxidative stress and finally autophagy.

## 2. Results

Experiments were carried out on 158N cells at 24 or 48 h of treatment in order to observe a real effect on the organelles and mechanisms involved in the toxicity of VLCFA. The concentrations used were the same of the highest used previously, these levels being similar to those encountered in patients and used in vitro in different models (fibroblasts, oligodendrocytes, mixed culture from rat brain hippocampus) [11,12,13].

### 2.1. Effects of DHA on Proliferation, Plasma Membrane, Mitochondria, and Oxidative Stress

Different tests were performed on 158N cells to assess cell proliferation: phase contrast microscopy, plasma membrane integrity with the use of propidium iodide (identification of cells with loss of plasma membrane integrity and/or dead cells), mitochondrial activity with the MTT test (based on succinate dehydrogenase activity) and oxidative stress with the use of DHE (measurement of reactive oxygen species (ROS) overproduction including superoxide anion overproduction).

**Figure 1:**
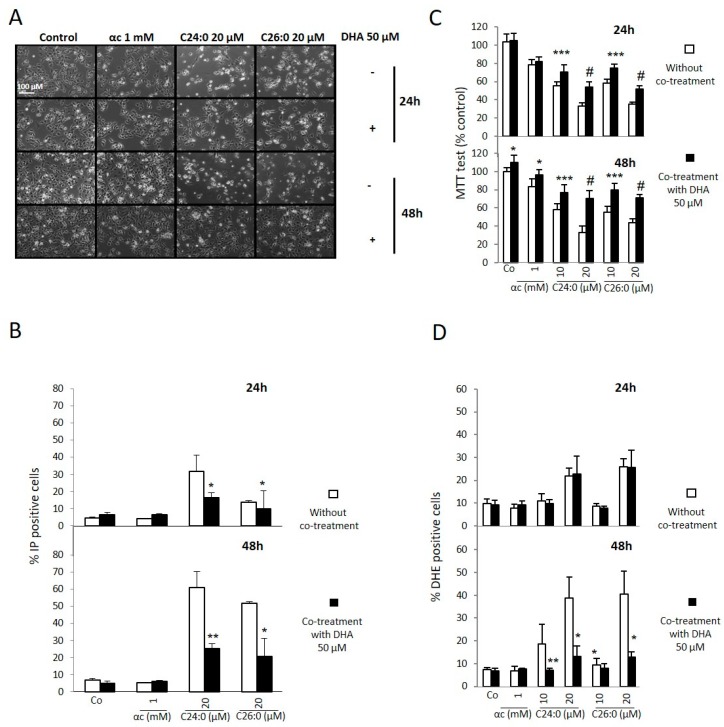
Effects of docosahexaenoic acid (DHA) on very long-chain fatty acids (VLCFA) (C24:0 and C26:0) toxicity in 158N murine oligodendrocytes. Murine oligodendrocytes were cultured with C24:0 or C26:0 at 10 or 20 µM in the presence or absence of DHA at 50 µM for 24 and 48 h. (**A**): The effect on cell growth, cell adhesion, and cell detachment was determined by phase contrast microscopy. (**B**): the effects of VLCFA and/or DHA on membrane integrity were studied by flow cytometry after staining with propidium iodide. (**C**): the effects of VLCFA and/or DHA on mitochondrial metabolism were evaluated with the MTT assay. (**D**): effects of DHA on the intracellular production of reactive oxygen species (ROS) (including superoxide anion (O_2_^−^)) induced by VLCFA at 24 and 48 h; evaluation by flow cytometry after staining with dihydroethidium (DHE). Statistical analysis with Mann-Whitney test: * *p* < 0.05; ** *p* < 0.01, *** *p* < 0.001, and # *p* < 0.0001.

The impact of VLCFA (C24:0 and C26:0) was morphologically assessed by phase contrast microscopy using the most toxic concentration (20 µM), as previously assessed at 24 and 48 h of treatment (Figure 1A), The cytoprotective effect of DHA (50 µM) was evaluated in C24:0- and C26:0-treated cells. For untreated cells at either 24 h or 48 h, the cells are adherent, spread out and not very refractive. In the case of VLCFA C24:0 and C26:0, the cells are less numerous, more refractive, and round for some cells at 24 h, with an amplified phenomenon at 48 h. When DHA (50 µM) is added in the presence of VLCFA, the cells are more numerous, and more adherent and spread out (Figure 1A).

The integrity of the plasma membrane was assessed using propidium iodide which is interspersed in the DNA only if the plasma membrane is permeable. In the case of VLCFA (20 µM) after 24 and 48 h, there is an increase in the number of propidium iodide positive cells (Figure 1B) and thus a permeabilization of the plasma membrane. With the use of DHA (50 µM), the number of propidium iodide positive cells decreases compared to VLCFA conditions alone. The addition of DHA maintains the integrity of the plasma membrane (Figure 1B).

Mitochondrial activity (to assess cell proliferation and/or viability) was measured with the MTT test at 24 and 48 h of treatment with VLCFA (10 and 20 µM) with or without DHA (50 µM) (Figure 1C). The results were expressed as a percentage of the control value. When VLCFAs are used alone, mitochondrial activity falls relative to control (Figure 1C). When DHA is used in co-treatment with VLCFA, mitochondrial activity is lower than the control but higher than with VLCFA alone. Mitochondrial activity is restored using DHA, indicating that there are more cells with functional mitochondria; this corroborates the data observed by phase contrast microscopy.

When the cells are treated with DHA, a similar percentage of DHE-positive cells are observed at 24 h. However, at 48 h, the effect of DHA is clearly visible. The cells recover almost totally to an oxidative level similar to that of the control cells (Figure 1D). The results observed with oxidative stress are obtained under conventional conditions of cell culture which transiently presents hyperoxia. Consequently, these results should be verified in models close to the conditions found *in vivo*, since cell cultures under these “conventional” conditions have certain limitations [14,15,16].

Altogether, our data show that VLCFA (C24:0 or C26:0) induce a type of cell death characterized by a decrease in cell count, a loss of plasma membrane integrity, a decrease in mitochondrial activity and an increase in oxidative stress. DHA attenuates the cytotoxic effects observed with VLCFA.

### 2.2. Effects of DHA on Autophagy Process

**Figure 2:**
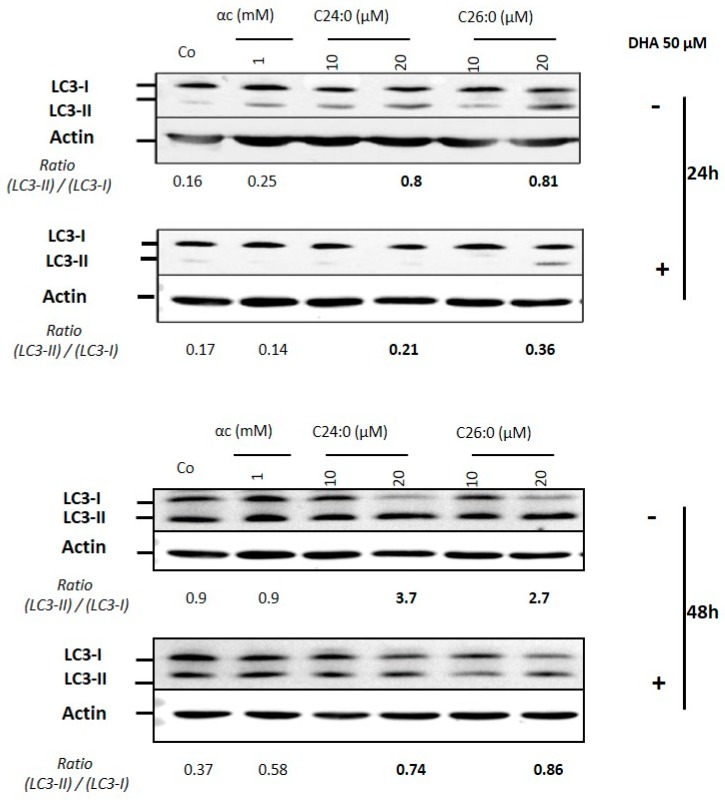
Effects of DHA on VLCFA (C24:0 or C26:0)-induced autophagy on 158N murine oligodendrocytes. Murine oligodendrocytes were cultured with C24:0 or C26:0 at 10 or 20 µM in presence or absence of 50 µM DHA for 24 and 48 h. Autophagy was evaluated by Western blotting, detecting the conversion of LC3-I to LCC3-II.

In previously published research, autophagy has been described as a protective process in cells, with a rescue that can be observed from 24/48 h onwards depending on the parameters studied [5]. If autophagy cannot allow the cells to resist the toxicity of VLCFA, cells can develop apoptosis and/or necrosis. We, therefore, evaluated whether the beneficial effects of DHA observed on the previous parameters (viability, plasma membrane permeability, mitochondria, oxidative stress) were also observed at the autophagic level. By immunoblotting, we studied the effect of DHA on the status of LC3 protein. During the elongation stage of the autophagic process, the LC3 protein is cleaved (LC3-I form) and conjugated to phosphatidylethanolamine (LC3-II form). The LC3-II form, found on both sides of the autophagosome membrane, is a marker of autophagy. The presence of LC3-I and LC3-II forms was assessed, as well as the ratio LC3-II/LC3-I (Figure 2).

When used alone, C24:0 or C26:0, induce an increase of the LC3-II form, especially at concentrations of 20 µM. This can be seen through the increase in LC3-II/LC3-I ratio from 0.16 for control cells to 0.8 on average for (20 µM) C24:0 and C26:0 at 24 h of treatment; and from 0.9 in control cells to 3.7 or 2.7 for C24:0 and C26:0, respectively, at 48 h. These ratio variations are consistent with those presented in the literature for the study of this protein. When cells are treated with VLCFA and DHA (50 µM), either at 24 h or 48 h, there is a decrease in the presence of the LC3-II form. Similarly, the LC3-II/LC3-I ratio, which was 0.8 on average for C24:0 or C26:0 at 24 h, drops to 0.21 and 0.36 in the presence of DHA. At 48 h, similar changes are observable with a ratio that drops from 3.7 and 2.7 for C24:0 and C26:0, respectively, to 0.74 and 0.86 in the presence of DHA with C24:0 or C26:0, respectively (Figure 2). DHA, by reducing the toxicity of VLCFA, also reduces autophagy.

This also confirms that VLCFA-induced autophagy can be considered a protective mechanism, as previously demonstrated by our team the laboratory in the same cell model and by another team on neurons [5,17].

## 3. Discussion

To treat neurodegenerative diseases, one strategy may be to modify nutrition by promoting certain foods rich in nutrients of interest, or by adding food supplements, thus developing functional food or nutraceutical strategies. The challenge in the therapeutic use of food is that the delivery of molecules of interest to the place where the pathological process develops. Many studies have thought to act on oxidative stress, since it is a common and quite early event in the development of different neurodegenerative diseases. However, at present, no therapy based on antioxidants is routinely used. This is why we have turned to the use of molecules present in food and in particular DHA. In the context of peroxisomopathies, where an accumulation of VLCFA is observed, it is necessary to better understand the mechanisms, but also to identify molecules capable of improving patients’ condition. Since the accumulation of VLCFA causes oligodendrocyte cell death, we used a mouse model of oligodendrocytes, 158N cells treated with C24:0 or C26:0 at concentrations found in patients. It is recognized that plasma membrane changes, mitochondrial dysfunction and oxidative stress occur during VLCFA-induced toxicity. During this toxicity, we also showed that the cells put in place a protective autophagic process, and in the event that the damage was too great, they entered into either apoptotic or necrotic cell death. The purpose of this study was to assess the ability of DHA to counteract the effects of VLCFA. Effects on cell viability, plasma membrane, mitochondria, and oxidative stress were assessed. We have shown that DHA is capable of restoring cell viability, plasma membrane integrity, and mitochondrial functionality. This is accompanied by a decrease in oxidative stress and by a normalization of autophagic process which contributes to the decrease in oligodendrocyte cell death following treatment with VLCFA. Currently, DHA has been described to prevent Alzheimer’s disease. A neural model study (SK-N-BE cells) showed that DHA was able to inhibit the toxic effects of VLCFAs at the mitochondria level and on cell growth, regardless of the concentration of DHA used, while oxidative stress reduction was only observed at low concentrations (50 µM) [10]. Two other studies showed that DHA was able to counteract the toxic effects of certain oxidized cholesterol derivatives (oxysterols), which are produced during the development of various pathologies, mainly age-related diseases, in the 158N oligodendrocyte model and in the SK-N-BE cell neuron model [10,18]. Thus, DHA is a molecule that could be added to the therapeutic arsenal currently available to patients with neurodegeneration.

This pilot study showed that DHA counteracts the cytotoxic effects of VLCFA. DHA attenuates plasma membrane permeability, mitochondrial dysfunction, and oxidative stress, and normalizes autophagy. Additional studies are necessary to validate and complete these results *in vitro*, but also in vivo. In the case of DHA, a clinical trial was conducted and showed that its use as a dietary supplement did not give positive results in individuals with peroxisome assembly disorders [19]. Taking this into account, consideration should be given to another way of bringing DHA to the brain, by moving towards functionalization or by combining it with other products as has been the case with Lorenzo’s oil [20,21]. Indeed, the brain is protected by the blood-brain barrier both physically (close control of exchange between the blood and the brain compartment) and metabolically (intra- and extracellular expression of enzymes such as monoamine oxidase (MAO) or γ-glutamyl transpeptidase (γ-GT), capable of hydrolyzing substrates that may be toxic to the Central Nervous System (CNS)). It is also protected by the presence of efflux pumps such as P-gp (P-glycoprotein), MRP (multidrug resistance-associated proteins) or BCRP (Breast Cancer Resistant Protein): proteins expressed by brain endothelial cells, which prevent certain substances from reaching the CNS or which facilitate their elimination in the brain. This barrier therefore governs the access of drugs and other molecules to the CNS. In order to place nutrients as a therapeutic option in neuropharmacology, several questions will arise. Will the compounds be able to reach the CNS? Are there any other options than supplementing the diet? One of the answers may lie in nanotechnologies or systems for diffusing molecules into the nose.

## 4. Materials and Methods

### 4.1. Cell Culture

Murine oligodendrocytes cells (158N) were cultured in Dulbecco’s Modified Eagle Medium (DMEM) (Lonza, Amboise, France) supplemented with 5% (*v*/*v*) heat-inactivated fetal bovine serum (Dutscher, Brumath, France) and 1% antibiotics (penicillin, streptomycin) (Dutscher). Cells were seeded at 5000–10,000 cells/cm^2^ either in Petri dishes (100 mm in diameter), or 12-well plates. The cells were incubated at 37 °C in a humidified atmosphere containing 5% CO_2_ and under atmospheric pO_2._ Cells were trypsinized (0.05% trypsin−0.02% EDTA solution), and passaged twice a week.

### 4.2. Cell Treatment

C24:0 (tetracosanoic acid) and C26:0 (hexacosanoic acid) were from Sigma–Aldrich (St. Louis, MO, USA). The VLCFA were solubilized in α-cyclodextrin (Sigma–Aldrich) as previously described [22]. Final concentration of α-cyclodextrin (vehicle) in the culture medium is 1 mM for the highest concentration used in VLCFA [11]. The cells were further treated for 24 h and/or 48 h with various VLCFA concentrations (10 and 20 µM) and with α-cyclodextrin (1 mM) in HAM’s-F10 medium (medium containing no long-chain fatty acid).

Docosahexaenoic acid (DHA) (Sigma–Aldrich) was prepared at 8 mM in α-cyclodextrin (20 mM), conserved at 4 °C and added in the culture medium at 50 µM with or without VLCFA.

### 4.3. Analysis of Cell Morphology by Phase Contrast Microscopy

Cell morphology was observed after 24 h and 48 h of treatment, in the absence or presence of VLCFA (20 µM), α-cyclodextrin (1 mM) or DHA (50 µM) under an inverted-phase contrast microscope (Axiovert 40CFL, Zeiss, Jena, Germany). Digitized images were obtained with a camera (Axiocam ICm1, Zeiss).

### 4.4. Colorimetric MTT Assay

MTT assay (Sigma–Aldrich) was carried out on oligodendrocytes after 24 h or 48 h of treatment with C24:0 or C26:0 (10, and 20 µM), α-cyclodextrin (1 mM) or DHA (50 µM) as described previously. The MTT assay was used to evaluate the effects of DHA on cell proliferation and/or viability changes induced by C24:0 or C26:0. MTT salt is reduced to formazan in the metabolic active cells by dehydrogenase to form NADH and NADPH [23]. The plates were read at 570 nm with a microplate reader (TECAN Sunrise, Tecan, Lyon, France).

### 4.5. Flow Cytometric Measurement of Cell Viability with Propidium Iodide

Propidium iodide was used to determine cell viability (PI; λEx_max_ = 540 nm, λEm_max_ = 625 nm, Sigma) (1 µg/mL). PI binds to DNA by intercalating between the bases only if the plasma membrane is damaged as is the case for dead cells [24]. The red fluorescence was quantified by flow cytometry in 10,000 cells on a logarithmic scale of fluorescence with a Galaxy flow cytometer (Partec, Münster, Germany) by using a 630 nm longpass filter. Data were analyzed with FlowMax software (Partec).

### 4.6. Flow Cytometric Measurement of ROS Overproduction with Dihydroethidium

ROS overproduction, including superoxide anion (O_2_.^−^), and hydroxyl radical, was detected with dihydroethidium (DHE; λEx_max_ = 518 nm, λEm_max_ = 605 nm, Life Technologies, St Aubin, France) [25,26]. DHE diffuses through cell membranes, and is rapidly oxidized into ethidium under the action of ROS. DHE (1.6 mM, dimethyl sulfoxide (DMSO) (Sigma–Aldrich)) was used at 2 μM. After 15 min at 37°C, the fluorescent signals of DHE stained cells were collected through a 590/10 nm on a logarithmic scale on a GALAXY flow cytometer (Partec); 10,000 cells were acquired; data were analyzed with Flomax (Partec) software.

### 4.7. Protein Analysis by Polyacrylamide Gel Electrophoresis and Western Blotting

Cells were lysed in a lysis buffer (120 mM Tris-HCl, pH 6.8, 1.4% SDS) in the presence of 1/25 complete protease inhibitor cocktail tablets (Roche Diagnostics Corporation, Indianapolis, IN, USA) for 30 min on ice. Cell lysates were cleared by a 15 min centrifugation at 20,000× *g*. The Bicinchoninic Acid Solution (Sigma-Aldrich) was used to determine protein concentration. Eighty micrograms of proteins were diluted in loading buffer (20% glycerol, and 0.02% bromophenol blue), separated on a polyacrylamide SDS-containing gel, and transferred onto a nitrocellulose membrane (Thermo-Scientific, Waltham, MA, USA). Nonspecific binding sites were blocked for 1h with 5% nonfat milk in TBST (10 mM Tris-HCl, 150 mM NaCl, 0.1% Tween 20, pH 8), the membrane was incubated overnight with the primary antibody (TBST with 1–5% milk). The antibodies directed against LC3-I/II (L8918) were from Cell Signaling (Ozyme, Saint Quentin Yvelines, France), and used at 1/1000 final concentration. An antibody directed against β-actin (Sigma-Aldrich) was used at 1/10,000 final concentration. After three washes with TBST, membrane was incubated (1 h, room temperature) with horseradish peroxidase-conjugated goat anti-rabbit antibody (Cell Signaling) diluted at 1/5000. The membrane was washed with TBST and revealed using an enhanced chemiluminescence detection kit (Supersignal West Femto Maximum Sensitivity Substrate, Thermo-Scientific) and Chemidoc XRS+ (Bio-Rad, Marnes la Coquette, France). The ratio LC3-II/LC3-I was calculated with Image Lab software (Bio-Rad).

## 5. Conclusions

Altogether, these data support that DHA remains a promising therapeutic molecule but its administration for neurodegenerative diseases still needs to be developed. Indeed, innovative solutions in research and development will be necessary so that molecules derived from food or metabolism can be used as therapeutic tools.

## Abbreviations

BCRP	breast cancer resistant protein
CNS	central nervous system
DHA	Docosahexaenoic acid
DHE	dihydroethidium
DMSO	dimethylsulfoxide
γ-GT	γ-glutamyl transpeptidase
MAO	monoamine oxidase
MRP	multidrug resistance-associated proteins
P-gp	P-glycoprotein
PI	propidium iodide
VLCFA	very long-chain fatty acid
